# Lipid Liquid Crystal Nanoparticles: Promising Photosensitizer Carriers for the Treatment of Infected Cutaneous Wounds

**DOI:** 10.3390/pharmaceutics15020305

**Published:** 2023-01-17

**Authors:** Muhammed Awad, Zlatko Kopecki, Timothy J. Barnes, Anthony Wignall, Paul Joyce, Nicky Thomas, Clive A. Prestidge

**Affiliations:** 1 Centre for Pharmaceutical Innovation, University of South Australia, Clinical and Health Sciences, Adelaide, SA 5000, Australia; 2 Pharmaceutical Analytical Chemistry Department, Faculty of Pharmacy, Al-Azhar University, Assiut 71524, Egypt; 3 Future Industries Institute, STEM Academic Unit, University of South Australia, Mawson Lakes, SA 5095, Australia

**Keywords:** chronic wounds, antimicrobial, photodynamic therapy, liquid crystal nanoparticles

## Abstract

Cutaneous chronic wounds impose a silent pandemic that affects the lives of millions worldwide. The delayed healing process is usually complicated by opportunistic bacteria that infect wounds. *Staphylococcus aureus* is one of the most prevalent bacteria in infected cutaneous wounds, with the ability to form antibiotic-resistant biofilms. Recently, we have demonstrated the potential of gallium protoporphyrin lipid liquid crystalline nanoparticles (GaPP-LCNP) as a photosensitizer against *S. aureus* biofilms in vitro. Herein, we investigate the potential of GaPP-LCNP using a pre-clinical model of infected cutaneous wounds. GaPP-LCNP showed superior antibacterial activity compared to unformulated GaPP, reducing biofilm bacterial viability by 5.5 log_10_ compared to 2.5 log_10_ in an ex vivo model, and reducing bacterial viability by 1 log_10_ in vivo, while unformulated GaPP failed to reduce bacterial burden. Furthermore, GaPP-LCNP significantly promoted wound healing through reduction in the bacterial burden and improved early collagen deposition. These findings pave the way for future pre-clinical investigation and treatment optimizations to translate GaPP-LCNP towards clinical application.

## 1. Introduction

Antimicrobial resistance (AMR) is a major global threat that claims millions of lives and costs the global economy trillions of dollars annually [1]. One of the strategies that microbes use to escape host immune cells and conventional antibiotics is to live as aggregated colonies within self-produced protective matrices known as biofilms [2]. Biofilms are highly tolerant to antibiotics, and they are associated with 80% of infections, including those observed in chronic wounds [3,4]. Chronic wounds are characterized by delayed wound-healing responses, chronic unresolving inflammation and impaired host response to wound-localized infections which can quickly progress to sepsis [5,6]. The wound healing process can be divided into four phases; (1) haemostasis, (2) inflammation, (3) proliferation, and (4) remodelling [7]. Wound development and chronicity is often a secondary consequence of systemic conditions including obesity and/or diabetes mellitus, and is further compounded by pathogen colonization, biofilm formation and subsequent development of a localized clinical infection [8].

*Staphylococcus aureus* is one of the most abundant pathogenic bacteria on the skin, which is highly resistant to antibiotics and highly censured for wound infections [9]. Biofilms exhaust the host immune system by releasing planktonic cells as part of their life-cycle, further inducing wound chronicity and promoting the development of localized clinical wound infection [10]. Furthermore, biofilm infection “locks” the wounds in a prolonged inflammatory state via continuous production of enterotoxins and necrotizing substances to acquire nutrients from the host tissue, hence contributing to increased wound size, maceration, inflammation, and impaired healing. 

The high tolerance of biofilms to conventional antibiotics has urged on the investigation of unconventional treatment options, one of which is antimicrobial photodynamic therapy (aPDT) [11,12]. The term photodynamic therapy was first used by Von Tappeiner to describe the interaction between light, non-toxic dyes known as photosensitizers and oxygen to generate highly reactive oxygen species (ROS) [12]. Following the success of PDT in controlling skin cancer and precancerous lesions, including actinic keratosis [13,14], it has recently been investigated as a treatment option for chronic wounds [15] and has been shown to improve healing in clinical studies conducted on infected chronic skin ulcers [15]. 

The improved wound healing following aPDT treatment has been attributed to several mechanisms, including enhanced fibroblasts proliferation via upregulating mitochondrial activity, which increases collagen production and the synthesis of nucleic acid [16,17,18,19], and most importantly, reduces the bacterial burden caused by antibiotic-resistant strains [16,20]. Despite the promising results in the literature, the reported antibacterial activity of aPDT in vivo was often modest [20], and usually lower than the reported results in vitro [12,21]. The lower antibacterial activity in vivo is ascribed to the more complex behavior of biofilms within the wounds environment to escape host immunity [10]. Thus, more investigations using novel photosensitizers and thorough optimization of treatment parameters, i.e., light dose and photosensitizer concentration in ex vivo models, are necessary to enable the clinical translation of aPDT to manage infected chronic wounds.

Previously, we have demonstrated the potential of lipid liquid crystalline nanoparticles (LCNP) to promote the antimicrobial photodynamic activity of gallium protoporphyrin (GaPP) against the notorious antibiotic-resistant bacteria *S. aureus* and *Pseudomonas aeruginosa* [17,22]. LCNP-promoted singlet oxygen production from photoactivated GaPP eradicated *S. aureus* biofilms and increased the proliferation of human skin fibroblasts in vitro [17]. Therefore, we hypothesized that GaPP-LCNP could be applied in vivo to manage *S. aureus*-infected cutaneous wounds. To this end, we optimized GaPP-LCNP concentration and light dose in an ex vivo model before testing the antibacterial activity in full thickness excisional murine wounds to pave the way for future pre-clinical and clinical trials.

## 2. Materials and Methods

### 2.1. Materials

Monoolein (Myverol 18–92 K, Kerry ingredients) was received as a donation from DKSH Performance Materials Australia. Pluronic F127, propylene glycol and methanol were purchased from Sigma-Aldrich (St. Louis, MO, USA). Gallium protoporphyrin was obtained from Frontier Scientific (Logan, UT, USA). 

### 2.2. Preparation of GaPP-LCNP

The nanoparticles dispersion was prepared as previously reported [17,23]. Briefly, monoolein (15 mg), pluronic F127 (3 mg) and 260 µL propylene glycol were mixed with 0.5 mL GaPP methanolic solution (1.5 mM). Excess hydrotrope (methanol) was added, and the mixture was homogenised via vortexing for 2 min. Under a stream of N_2_ gas, a thin dry film was formed, which was dispersed using 5 mL of MQ water and sonication for 10 min.

### 2.3. Characterization of Nanoparticles Diameter

The average particle diameter (Z-average), zeta potential and polydispersity index (PDI) were determined using a Zetasizer Nano ZS (Malvern, Worcestershire, UK), as previously described [22].

### 2.4. Determination of GaPP Concentration

The concentration of GaPP within LCNP was determined using the native fluorescent signal of GaPP at 585 nm after excitation at 405 nm [22] using a Fluostar^®®^ Omega microplate reader. To determine the loaded GaPP concentration, a GaPP-LCNP dispersion in water was centrifuged for 10 min at 31,120 g; the unloaded GaPP precipitated, while the supernatant containing GaPP loaded in LCNP was dissolved in methanol, and the concentration was quantified from the corresponding calibration curve. A linear calibration curve in the range of (0.3–3 µM) with a correlation coefficient (r) of 0.9998 was established and used for determination of GaPP concentration. 

### 2.5. Spectrosocpic Studies

The fluorescence intensities of GaPP in different matrices were recorded using a Cary Eclipse Fluorescence Spectrometer. Briefly, the fluorescence intensity spectra of equimolar concentrations of GaPP (0.3 µM) dissolved in methanol, MQ water containing 1% DMSO and loaded in LCNP, were recorded after excitation at 405 nm.

### 2.6. Illumination Set Up 

The light source used for the photoactivation process is a mounted blue LED at 405 nm (M405L4) [22]. The light beam was collimated using an aspheric condenser lens attached to the mounted LED using an SM1 Lens Tube to illuminate an area of 1 cm. A T-Cube LED Driver, 1200 mA Max Drive Current (LEDD1B) was used to control the output power. The output power was monitored using a PM100USB power meter connected to a S302C thermal sensor head, all purchased from Thorlabs (Newton, NJ, USA).

### 2.7. Antibiofilm Activity of GaPP-LCNP In Vitro 

A single colony of *S. aureus* (Xen-29) was incubated in tryptic soy broth (TSB) containing 200 µg/mL kanamycin for 18 h at 37 °C. The bacterial suspension was adjusted to 0.5 McFarland followed by 1:100 dilution in kanamycin-supplemented TSB. A total of 100 µL of bacterial suspension was inoculated in 96-well plates for 24 h under static conditions to allow biofilm formation. Following biofilm establishment, unattached cells were removed via washing with saline, and 100 µL of GaPP or GaPP-LCNP was incubated with biofilms for 30 min in the dark; then, blue LED light [17] with a total irradiance of 0.267 Wcm^−2^ was used for photoactivation. After treatment, the biofilms were extracted using sterile pipette tips, followed by serial dilution and plating on kanamycin-supplemented tryptic soy agar for CFU enumeration.

### 2.8. Study of GaPP-LCNP Distribution in Biofilms In Vitro

*S. aureus* biofilms were established in an 8-well slide chamber as previously detailed [17]. Following biofilm formation, 200 µL of GaPP-LCNP with a final GaPP concentration of 50 µM was incubated with biofilms at 37 °C for 2 h. Following incubation, GaPP-LCNP were removed and biofilms washed to remove non-adherent nanoparticles. The biofilms were stained with Syto-9 (Eugene, OR, USA) for 15 min, followed by washing and fixation using neutral formalin. After removal of the fixative solution, the biofilms were washed and dried. The biofilms attached to the glass slide were examined using confocal microscopy (LSM800, Zeiss, Oberkochen, Germany) using a 10× objective lens. The live cells within biofilms stained with Syto-9 were observed at 525 nm following excitation at 507 nm, while GaPP fluorescent signal was monitored at 585 nm following excitation at 405 nm.

### 2.9. Antibiofilm Activity in an Ex Vivo Infection Model

Excisional wounds on the back of Balb/c mice (*n* = 6) were performed using a 6 mm biopsy punch. The wounds were covered with a Tegaderm^TM^ 3M dressing immediately following surgery. After 24 h, 10 µL containing 1.5 × 10^7^ CFU of the bioluminescent strain *S. aureus* Xen29 inoculum in PBS were applied to the wounds. Following infection, wounds were covered with a new Tegaderm^TM^ dressing to promote biofilm formation; the dressings were changed daily until the end of the study at day six. The progression of infection was monitored daily using an in vivo imaging system (IVIS) spectrum (PerkinElmer, Waltham, MA, USA) as previously described [24]. On day six, the mice were humanely killed, and the wound beds were collected, cut in half and used for ex vivo studies. 

The infected tissues were incubated with unformulated GaPP 50 µM, GaPP in LCNP with a final GaPP concentration of 50 µM and PBS in Eppendorf tubes at 37 °C. After incubation for 30 min, the infected tissues were removed from the treatment solution and exposed to blue light irradiance of 0.267 W/cm^2^ for 10 min. Following light treatment, the tissues were placed in fresh Eppendorf tubes containing 1 mL of sterile PBS and vortexed for 5 min to extract bacteria from the tissues followed by serial dilution and CFU enumeration. 

### 2.10. Efficacy Study of GaPP-LCNP in a Chronic Infected Wound in Mice 

Excisional wounds on the back of male and female Balb/c mice (*n* = 18) were performed using a 6 mm biopsy punch; the wounds were immediately covered with Tegaderm^TM^ 3 M following surgery. The mice were allowed to recover from surgery for 24 h, then the wounds were infected with 1.5 × 10^7^ CFU/mL of *S. aureus* Xen29 corresponding to an IVIS reading of 2 × 10^6^ (photons/s) and covered with a new Tegaderm^TM^ dressing to promote formation of mature biofilms. The bacterial growth was monitored using an IVIS spectrum in vivo imaging system, and biofilm formation was confirmed after 24 h, with observation of pus on wounds correlating with an IVIS reading of 2 × 10^7^ (photons/s). After establishment of biofilms on wounds, mice were allowed to rest for 24 h. On day three of infection, the dressings were removed, and mice were divided randomly into three groups; each group was treated with 20 µL of (1) unformulated GaPP (50 µM), (2) GaPP-LCNP (50 µM) or sterile saline. After 30 min of GaPP or GaPP-LCNP application, the wounds were exposed to blue LED light of 0.267 W/cm^2^ for 10 min. The treatment was repeated for three consecutive days. On day six, mice were sacrificed and wound beds were collected for CFU enumeration and histology studies following established protocols [25]. 

### 2.11. Wound Healing Assessment 

Wounds were photographed daily throughout the study from day zero to day six in order to observe the changes in wound morphology and assess wound healing. The wound area was determined as an indication of wound healing using Image J software as previously described [6]. Moreover, microscopic analysis of wounds was conducted after histology assessment. Briefly, wounded skin sections (4 µm) were cut on a rotary microtome (RM2235, Leica, Germany) and mounted onto glass super frost microscope slides (Menzel Gläser, Germany) for routine hematoxylin and eosin (H&E) staining [26]. Masson’s trichrome staining was used to evaluate the collagen content of the wounds by measuring color intensity following established protocol [27]. The slides were scanned using the Nanozoomer (Hamamatsu Photonics, Japan; 40× magnification) and visualized with Nanozoomer Digital Pathology software view.2 (NDP view v1.2; Histalim, France).

## 3. Results and Discussion 

### 3.1. Preparation and Characterization of Dispersible GaPP-LCNP

**Figure 1:**
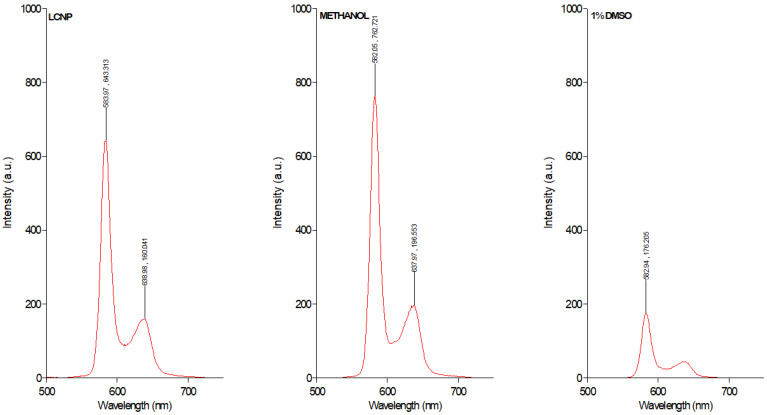
Fluorescence intensity of GaPP 0.3 µM dissolved in LCNP, methanol and aqueous solution containing 1% DMSO.

LCNPs are a unique class of nanomaterials that are prepared from the self-assembly of amphiphilic lipids in water to yield 3D crystalline nanoparticles [28]. In this study, glycerol monooleate was utilized as the amphiphilic lipid to fabricate LCNP using the hydrotrope dilution method as previously described [17,22]. This formulation approach produces uniformly dispersed LCNP with higher stability, due to uniform distribution of the stabilizer [28]. Herein, the obtained GaPP-LCNP dispersion has a Z-average diameter of 178 ± 3.1 nm with a polydispersity index of 0.19 ± 0.04, see Table 1. The 3D crystalline structure of LCNP with a high internal surface area [29] allows uniform distribution of GaPP molecules within the lipid bilayer with an entrapment efficiency of 98.3 ± 4.3%. The entrapment of GaPP in LCNP efficiently solubilizes GaPP in aqueous dispersion and keeps it in a monomeric form, and prevents the formation of aggregated species (GaPP)_2_ and (GaPP)_3_ usually formed in aqueous media [30]. The poor solubility of protoporphyrin derivatives such as GaPP results in the formation of aggregates and vesicles in aqueous solutions via π-π stacking [31]. These aggregates reduce the intersystem crossing between the singlet- and the triplet-excited states of the photosensitizer electrons, thus reducing photon emission, which is a crucial step in the photoactivation process [31,32]. Aggregated photosensitizers are characterized by a lower absorption coefficient, lower singlet oxygen production and quenched fluorescence [32]. To study the potential of LCNP in promoting GaPP solubility and preventing aggregates’ formation, we compared the fluorescent intensity of equimolar concentrations of GaPP dissolved in LCNP, methanol and aqueous solution containing 1% DMSO, see Figure 1.

The fluorescence spectrum data demonstrate strong quenching of GaPP fluorescence peaks at 583 nm and 638 nm in GaPP dissolved in aqueous solution containing 1% DMSO, due to aggregates’ formation. On the other hand, the fluorescent peaks of GaPP dissolved in LCNP resemble those of GaPP dissolved in methanol, where GaPP occurs predominantly in the monomeric form [30], with slight reduction in the fluorescent intensity; this is ascribed to the difference in polarity between water and methanol [32]. These data agree with our previous reported results of the higher absorption coefficient of GaPP in LCNP ɛ = 57,983 M^−1^ cm^−1^ compared to unformulated GaPP ɛ = 19,689 M^−1^ cm^−1^ [22], and the higher singlet oxygen quantum yield (ϕ_∆_) of 0.72 compared to 0.42 with unformulated GaPP solution [17]. These data indicate the success of LCNP in solubilizing GaPP and keeping it in its monomeric form.

Improving the physicochemical characteristics of photosensitizers is a crucial step in promoting the clinical application of aPDT. Hydrophobic photosensitizers demonstrate strong photodynamic activity in organic solvents but fail to replicate this activity in biologically relevant aqueous solutions [33]. Although several formulations successfully solubilize hydrophobic photosensitizers, e.g., polymeric micelles and liposomes, concurrent improvement in the photodynamic activity is not always reported [12]. This is ascribed to the aggregation of photosensitizers within such nanoformulations [34,35]. However, the bicontinuous lipid bilayer of LCNP provides a higher surface area, which enables the three times higher loading capacity of hydrophobic compounds compared to lamellar liposomes [36]. We have previously optimized the loading level of GaPP within LCNP and found that 3.3 ± 0.3 *w*/*w*% was the optimum loading level for aPDT application, and that further loading of GaPP resulted in aggregation of GaPP molecules and correlated with lower ROS production ɸ_∆_ = 0.33 [17]. In addition to the improvement of GaPP photophysical characteristics, LCNP provides higher stability for GaPP molecules in aqueous media. This was implemented from the release pattern of GaPP molecules at physiological pH, where less than 1% of the loaded GaPP molecules were released from LCNP [17]. Meanwhile, 20% of the loaded GaPP was released after 2 h in response to the addition of bacterial lipase which is abundant in infected tissues [37]. This gives the formulation a triggerable release behavior and improves selectivity towards infected tissues. The facile fabrication of GaPP-LCNP with possible manufacture scale-up [28], in addition to the improvement of GaPP’s physicochemical characteristics, justifies investigating GaPP-LCNP as a potential formulation to be applied to infected chronic wounds in vivo.

### 3.2. Evaluation of Antibiofilm Activity In Vitro

**Figure 2:**
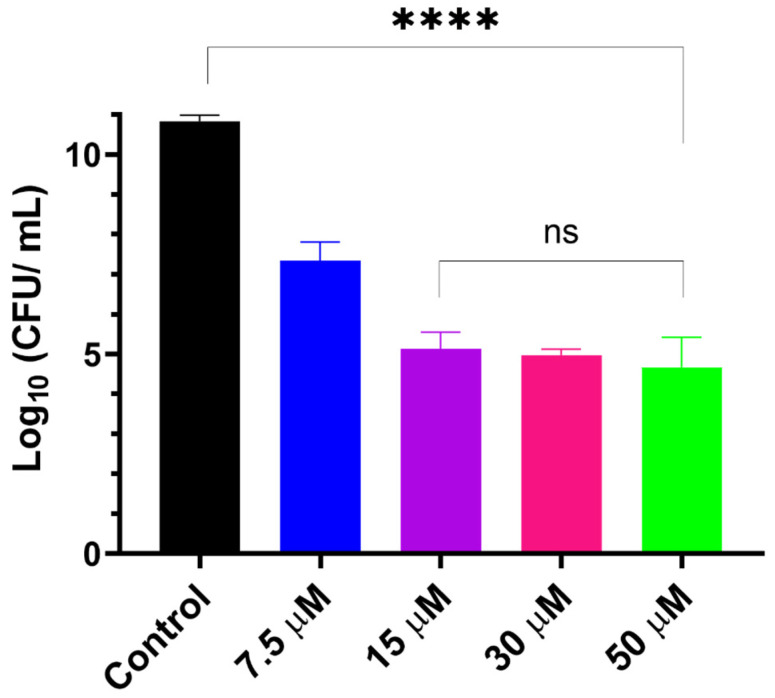
Viability of *S. aureus* biofilms following treatment with different concentrations of GaPP within LCNP, light dose 16 J/cm^2^. Data plotted as mean ± SD, *n* = 3. ns: non-significant, **** significant reduction in viability, *p* value < 0.0001 (one-way ANOVA test followed by multiple comparison Tukey’s test).

LCNPs have been shown to promote the antibiofilm activity of GaPP against different strains of *S. aureus* biofilms [17]. However, the difference in virulence factors among *S. aureus* strains changes the degree of complexity in the biofilm structure [38,39]. Thus, we anticipated that the bioluminescent *S. aureus* strain (Xen29) may behave differently from the previously tested standard strains 25,923 and USA 300 [17], and may therefore require optimization of the photoactivation parameters. We examined different concentrations of GaPP-LCNP against Xen29 biofilm in vitro before proceeding to the ex vivo model. Interestingly, 15 µM GaPP showed strong antibacterial activity, in agreement with our previous findings [17], reducing the viability of biofilms by ~6 log_10_ following one minute of light illumination with a total irradiance of 0.267 Wcm^−2^, see Figure 2. Further increase in GaPP concentration did not significantly increase the antibacterial activity. Thus, 15 µM GaPP-LCNP was considered the optimum concentration for further in vitro efficacy studies. 

Previously, we observed a decline in the antibacterial activity of GaPP-LCNP when the concentration of GaPP was increased above 3 µM against *P. aeruginosa* biofilms [22]. This was ascribed to the inactivation of GaPP molecules localized on biofilm boundaries by the generated ROS. However, in this study, the photobleaching of GaPP was less prominent in the *S. aureus* in vitro model. Thus, we anticipated that GaPP-LCNP diffused better through *S. aureus* biofilm matrix, contrary to our previous observation with *P. aeruginosa* biofilms [23].

**Figure 3:**
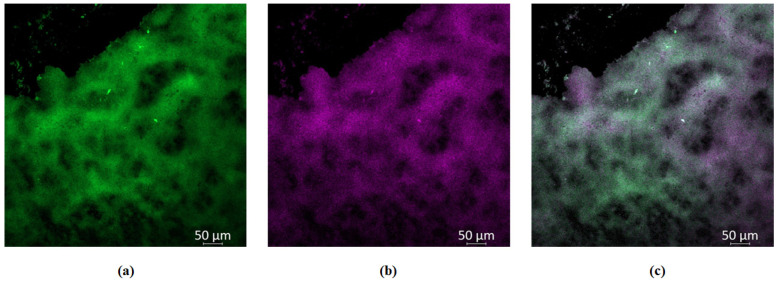
Confocal images of *S. aureus* biofilms showing wide distribution of GaPP within biofilms. (**a**) green channel showing viable *S. aureus* within biofilms stained with Syto-9 at 525 nm, (**b**) violet channel showing GaPP fluorescent signal at 585 nm, (**c**) mixed channel showing the distribution of GaPP within biofilms. The decline in Syto-9 fluorescent signal is attributed to the reduction in viability due to GaPP antibacterial activity.

To test this hypothesis of better GaPP diffusion through *S. aureus* biofilms further, we visualized the distribution of GaPP within biofilms using GaPP’s inherent fluorescence signal at 585 nm. The fluorescent signal of GaPP was detected in confocal imaging, see Figure 3, i.e., wide scattering of GaPP within the biofilm matrix. In contrast, in the *P. aeruginosa* biofilms, the fluorescent signal of GaPP could not be detected following the same incubation period (see Supplementary Figure S1). The distribution of GaPP-LCNP within *S. aureus* biofilms reduced the effect of photobleaching upon light activation, which was implemented from the non-significant change in the antibacterial activity of GaPP-LCNP at higher GaPP concentrations, see Figure 2, rather than a decline as seen with *P. aeruginosa* biofilms [22]. Furthermore, these images indicate the ability of LCNP to diffuse through the *S. aureus* biofilm matrix, since no signal was detected with the unformulated GaPP solution at the same concentration and incubation period.

**Figure 4:**
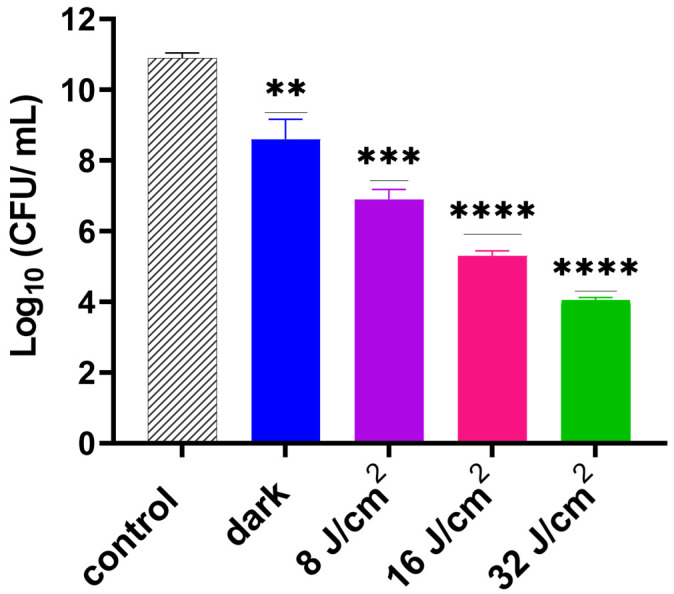
Viability of *S. aureus* biofilms following activation of GaPP-LCNP 15 µM using different light doses of blue light at 405 nm (8–32 J/cm^2^) compared to the negative control in the dark. Data plotted as mean ± SD, *n* = 3. ** significant reduction *p* = 0.0018, *** *p* = 0.0001, **** *p* <0.0001. (one-way ANOVA test followed by multiple comparison Dunnett’s test).

Following optimization of the GaPP dosing concentration, we examined the effect of different light doses on the photodynamic activity of GaPP-LCNP against biofilms. After 30 min incubation in the dark, GaPP-LCNP reduced the viability of *S. aureus* biofilms by 2.5 log_10_, presumably through the disruption of iron metabolic pathways as previously described [17]. Upon light activation, a significant reduction in biofilm efficacy was prominent, with a direct correlation between light dose and the antibacterial activity of GaPP-LCNP, see Figure 4. The highest antibacterial activity was recorded following light illumination for 2 min with total energy fluence of 32 J/cm^2^, where the viability was reduced by 7 log_10_; this confirms the positive impact of light dose on antibacterial activity.

**Figure 5:**
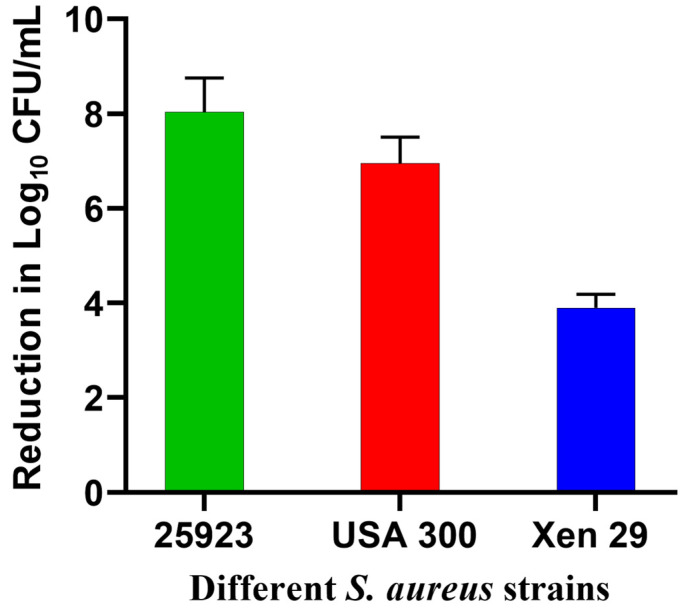
Reduction in the viability of different strains of *S. aureus* biofilms after incubation with GaPP-LCNP in TSB for 24 h in the dark, presented as log_10_ reduction in CFU count vs. different biofilm strains; data presented as mean ± SD.

In this study, Xen29 biofilm required a higher light dose compared to our previous report against standard strains of *S. aureus* biofilms, where only 3.8 J/cm^2^ were sufficient to eradicate biofilms [17]. This was partially attributed to the susceptibility of these tested biofilms to GaPP as an iron mimetic agent [40], in addition to the strong diffusion of GaPP-LCNP through the biofilm, which adds to the antibacterial activity upon light activation of GaPP [17]. To test whether Xen29 biofilms share the same susceptibility to GaPP as an iron mimic agent, we incubated GaPP-LCNP with the three *S. aureus* strains (25923, MRSA USA 300 and Xen29) for 24 h in the dark, followed by CFU enumeration. *S. aureus* 25,923 showed the highest susceptibility to GaPP-LCNP with an 8 log_10_ reduction followed by USA 300 7 log_10_ and Xen29 4 log_10_, see Figure 5. The lower susceptibility of Xen29 biofilms to GaPP-LCNP can be ascribed to the difference in virulence factors among *S. aureus* strains [39] which significantly affects the complexity of biofilms [41]; this explains the need for higher light doses to inactivate Xen29 biofilms.

**Figure 6:**
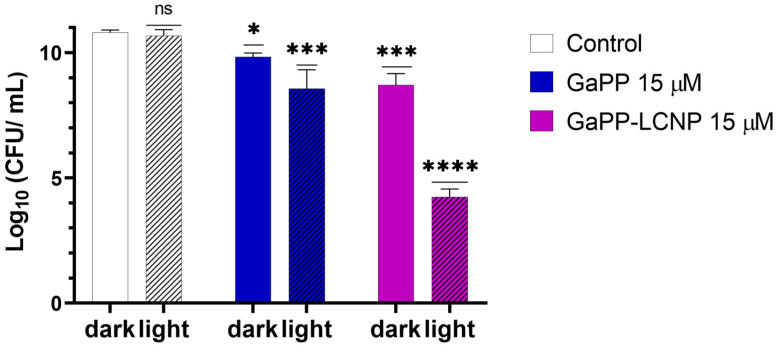
The viability of *S. aureus* (Xen29) biofilms following treatment with unformulated GaPP (15 µM) and GaPP-LCNP (15 µM), with and without blue light activation, energy fluence 32 J/cm^2^. Data plotted as mean ± SD, *n* = 3. ns: non-significant, * Significant reduction in viability *p* = 0.04, *** *p* = 0.0002, **** *p* < 0.0001 (two-way ANOVA test followed by multiple comparison Tukey’s test).

Despite the higher resilience of Xen29 biofilms to GaPP compared to previously tested strains [17], incorporating GaPP within LCNP significantly improved its antibacterial activity, see Figure 6. Following photoactivation with blue light, GaPP-LCNP reduced the viability of mature *S. aureus* biofilms by ~7 log_10_ compared to 2 log_10_ reduction by the unformulated GaPP solution. In addition, the antibacterial activity of GaPP in the dark was significantly enhanced; GaPP-LCNP reduced the viability of Xen29 biofilms by 2.5 log_10_ compared to 1 log_10_ by unformulated GaPP. 

Incorporating GaPP within LCNP and optimization of the light dose significantly reduced the viability of biofilms to the extent of eradication; there was a 7 log_10_ reduction in viability, compared to 8 log_10_ and 5 log_10_ for the standard strains 25,923 and USA 300, respectively. These findings emphasize the importance of optimizing different parameters affecting aPDT activity, and the ability of aPDT to overcome phenotypic resistance among different microbial strains.

### 3.3. Efficacy Study on Infected Skin Ex Vivo Model

**Figure 7:**
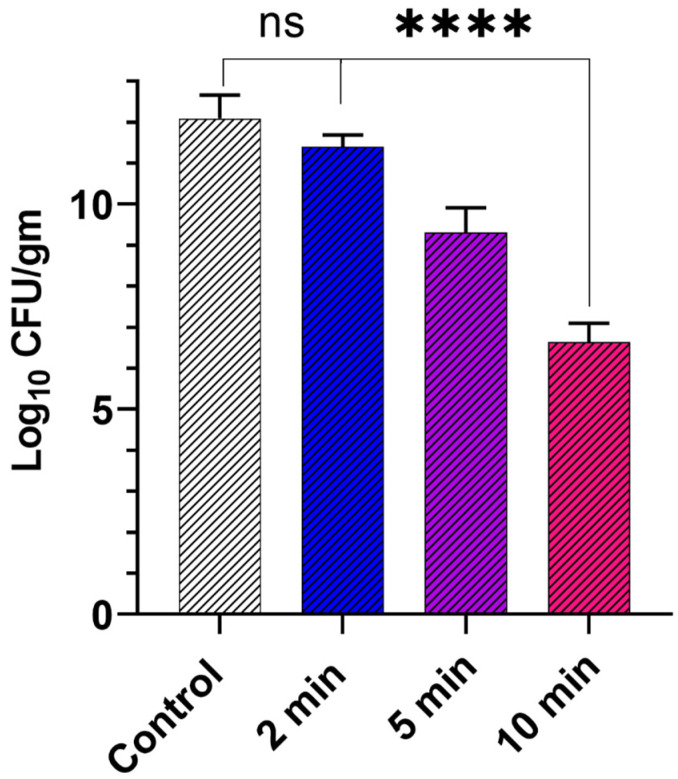
The viability of Xen29 biofilms collected from infected mouse skin wound after treatment with GaPP in LCNP 50 µM activated with blue light at different time intervals using 0.267 W/cm^2^ irradiance, compared to the control group incubated with PBS and illuminated with blue light for 10 min. Data presented as mean ± SD (*n*= 6, three technical replicates and 2 biological replicates); ns: non-significant, **** *p* value < 0.0001 (one-way ANOVA test followed by multiple comparison Dunnett’s test).

Before testing the activity of GaPP-LCNP in the murine model, we optimized vital parameters that influence the photodynamic activity of GaPP-LCNP against biofilms in an ex vivo model of chronically infected wounds, see Figure 7. The optimum conditions in vitro, 15 µM GaPP in LCNP and light activation for 2 min, reduced the biofilm activity by 0.9 log_10_. When GaPP concentration was increased to 50 µM at the same light dose, the biofilm activity was reduced by 1.4 log_10_ compared to the control group (Supplementary Figure S2). To improve the antibiofilm efficacy, we increased the illumination time while keeping irradiance at 0.267 W/cm^2^, and noticed a proportional reduction in the viability of biofilms upon increasing the illumination time.

The maximum reduction in viability was obtained after 10 min of tissue illumination, where the biofilms’ viability was reduced by 5.5 log_10_. Unlike in vitro studies, a photobleaching effect was not prominent in the ex vivo model despite the significant increase in light dose 160 J/cm^2^ and GaPP concentration 50 µM; which can be ascribed to the wide spatial distribution of GaPP molecules, where GaPP-LCNP is distributed between biofilms and the infected tissue and the absorption of blue light by tissue components which reduces the final light dose received by GaPP [42]. On the other hand, GaPP is more prone to photobleaching in the experimental set up in vitro, where photosensitizer molecules are confined in the small well area of the 96 well plate with negligible reduction in the received light dose, which increases the chance of photobleaching by the produced ^1^O_2_ at high photosensitizer concentrations [22,43]. 

**Figure 8:**
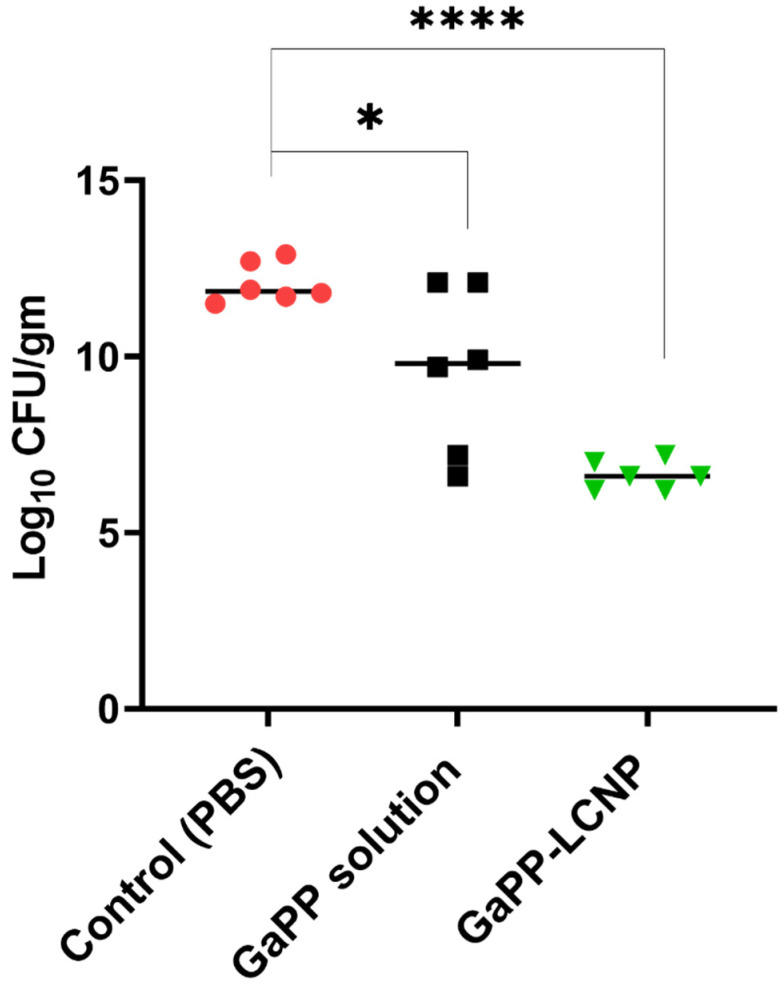
Viability of Xen29 biofilms incubated with PBS, GaPP or GaPP-LCNP 50 µM for 30 min in the dark followed by photoactivation for 10 min; (*n*= 6, three technical replicates and 2 biological replicates); * significant reduction *p* = 0.015; **** *p* < 0.0001(one-way ANOVA test followed by multiple comparison Dunnett’s test).

Thereafter, we compared the viability of GaPP-LCNP to unformulated GaPP solution using the same light dose and GaPP concentration, where GaPP-LCNP retained a superior antibacterial activity compared to unformulated GaPP with 5.5 log_10_ compared to 2.5 log_10_ with unformulated GaPP, see Figure 8. The difference in the activity between in vitro and ex vivo studies demonstrated the utmost importance of these studies to better elucidate the behaviour of photosenstizers in complex biological matrices and give realistic expectations of potential outcomes in animal studies. 

### 3.4. Efficacy Study in Infected Wounds In Vivo

**Figure 9:**
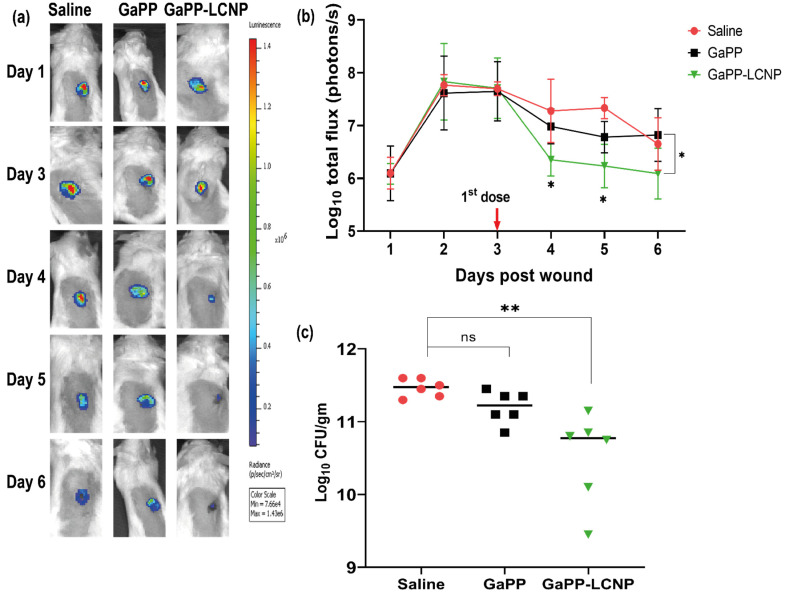
Illustration of the difference in the antibacterial activity between unformulated GaPP and GaPP-LCNP against Xen29 biofilms in infected full excisional wounds in mice. (**a**) Representative bioluminescent images showing infection progress in a single representative mouse from each treatment group; (**b**) corresponding determinations of the bacterial burden using average total flux (photons/s) showing significant reduction in the Xen29 luminescent signal following first treatment dose with GaPP-LCNP; (**c**) the viability of biofilms in wounds at the end point of the study after collection of tissue beds expressed as CFU/gm. Data presented as mean ± SD (three technical replicates for each wound per group, *n* = 6), ns: non-significant, * significant reduction *p* < 0.05, ** *p* = 0.001 (one-way ANOVA test followed by multiple comparison Dunnett’s test).

Following the success of GaPP-LCNP in significantly reducing the viability of Xen29 biofilms in the ex vivo model, we moved to test the antibacterial activity in vivo using a full thickness excisional wound model. Akin to our findings in the previously tested models, GaPP-LCNP performed better than unformulated GaPP and significantly reduced the viability of mature biofilms at earlier time points compared to the control group, while unformulated GaPP could not achieve a significant reduction in bacterial viability at all time points of the study, see Figure 9. The antibacterial activity of GaPP-LCNP was prominent after the first dose of treatment at day three, evidenced by a significant reduction in the luminescent signal of Xen29 at day four and day five (*p* < 0.05) compared to the saline group Figure 9b. Interestingly, at day six, the luminescence signal of Xen29 was significantly lower in GaPP-LCNP treated group compared to unformulated GaPP (*p* < 0.05), but was not significant compared to saline group. The current study suffered the limitation of relatively low mice population per group. Thus, future investigations with more mice per group are justified to gain more statistical significance between treatment and control groups. However, a CFU assay, which is considered to be the golden standard for evaluating antibacterial activity [44], indicated a significant 1 log reduction in the number of *S. aureus* colonies, while the unformulated GaPP group demonstrated no significant reduction in the bacterial burden compared to the saline-treated group, see Figure 9c. 

The complexity of in vivo models, where different tissue components have the capacity to absorb blue light [15], has been demonstrated to reduce the final light dose absorbed by the photosensitizer and lower the potential antibacterial activity of aPDT in vivo [20]. However, the higher singlet oxygen production by GaPP-LCNP, quick attachment of LCNP to bacterial cells [45] and the better diffusion of GaPP-LCNP through biological barriers [46] have increased the antibacterial activity of GaPP-LCNP compared to unformulated GaPP. In addition, the higher absorption coefficient of GaPP within LCNP 57,983 M^−1^ cm^−1^ compared to unformulated GaPP solution 19,689 M^−1^ cm^−1^ [22] allows more efficient utilization of blue light reaching GaPP molecules, thus resulting in higher ^1^O_2_ quantum yield and significant antibacterial activity in vivo, despite the complexity of the infected wound tissue. 

### 3.5. Effect of GaPP-LCNP on Wound Healing 

**Figure 10:**
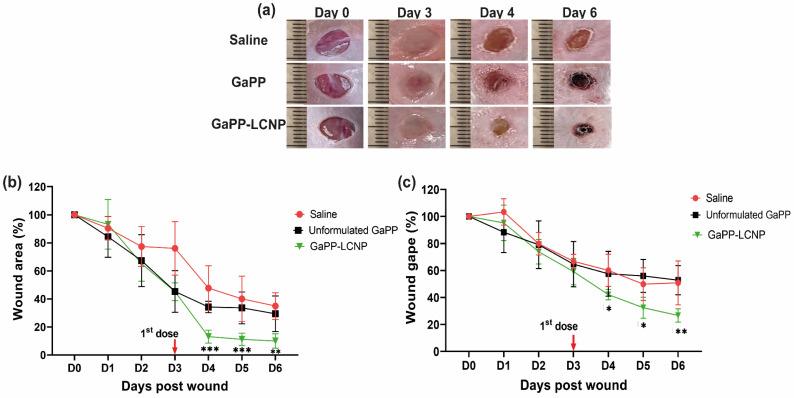
Monitoring the effect of GaPP-LCNP on infected wound healing. (**a**) Representative macroscopic images showing changes in wound morphology and wound staining with GaPP red colour; (**b**) wound-healing measurements obtained from the analysis of digital images presented as wound area % (**c**) Wound gape (%) during the study period; the wound size is expressed as 100% in the surgery day (D0) and the reduction in area and gape were calculated afterwards. Data presented as mean ± SD compared to control group treated with saline, * *p* < 0.05, ** *p* = 0.0001, *** *p* < 0.0001 (two-way ANOVA test, followed by multiple comparison Dunnett’s test).

One of the positive outcomes reported with the utilization of photodynamic therapy in the management of chronic wounds is the acceleration of wound healing [15,16,20]. We tested whether photoactivation of GaPP-LCNP would have a positive impact on the wound-healing process. Firstly, macroscopic analysis, which is a routinely used approach to determine the rate of wound healing, was conducted to determine wound size [20]. The wounds were imaged daily, followed by image analysis to determine the effect of the treatment on wound area and wound gape, following established protocols [25]. Figure 10a demonstrates the prominent changes in wound morphology during the study, including the surgery day (day 0) and day 3, where biofilm infections were established on the wounds showing pus and maceration; following the application of GaPP and GaPP-LCNP, wound maceration was ceased and the wounds appeared smaller in size with skin stain red in color due to GaPP color, as previously reported with other photosensitizers [47]. The GaPP-LCNP treated group demonstrated the highest wound healing with a ~90% reduction in wound area, see Figure 10b, compared to a 60% reduction with unformulated GaPP at day 4 post-wounding; this trend continued for remainder of the study. Additionally, a similar trend was observed when measuring wound gape, where GaPP-LCNP recorded the highest reduction in wound gape after the first dose (60%) and reached 75% at the end point of the study, showing significant improvement compared to controls. On the other hand, unformulated GaPP did not show a significant reduction in the wound gape compared to saline group, with ~60% wound gape at the study end point. 

**Figure 11:**
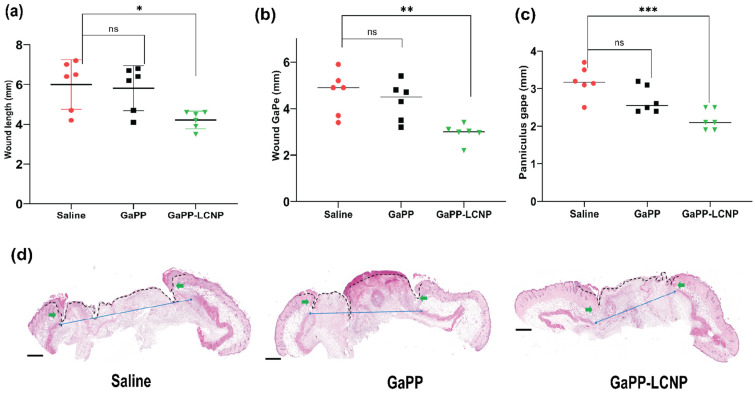
Microscopic analysis of the wound-healing process using histology analysis. (**a**) represents wound length, (**b**) wound gape and (**c**) panniculus gape; (**d**) representative histology section used for the obtained measurements; the dotted black line represents wound length, green arrows represent wound gape and blue arrows represent panniculus gape; magnification 10× H&E stain. Data presented as mean ± SD compared to control treated with saline; ns: non-significant, * *p* value = 0.01, ** *p* = 0.002 and *** *p* = 0.0003 (one-way ANOVA test followed by multiple comparison Dunnett’s test).

To achieve deeper insight on treatment efficacy on wound healing, we performed a histology analysis following animal sacrifice at the end of the study. Histological assessment confirmed the significant reduction in wound size with GaPP-LCNP compared to unformulated GaPP solutions, which recorded similar values to the untreated saline group, see Figure 11. The accelerated wound closure with GaPP-LCNP is believed to be correlated to the reduction of bacterial burden, see Figure 9, which exacerbate tissue damage through necrotizing substances and toxins [10,48]. 

**Figure 12:**
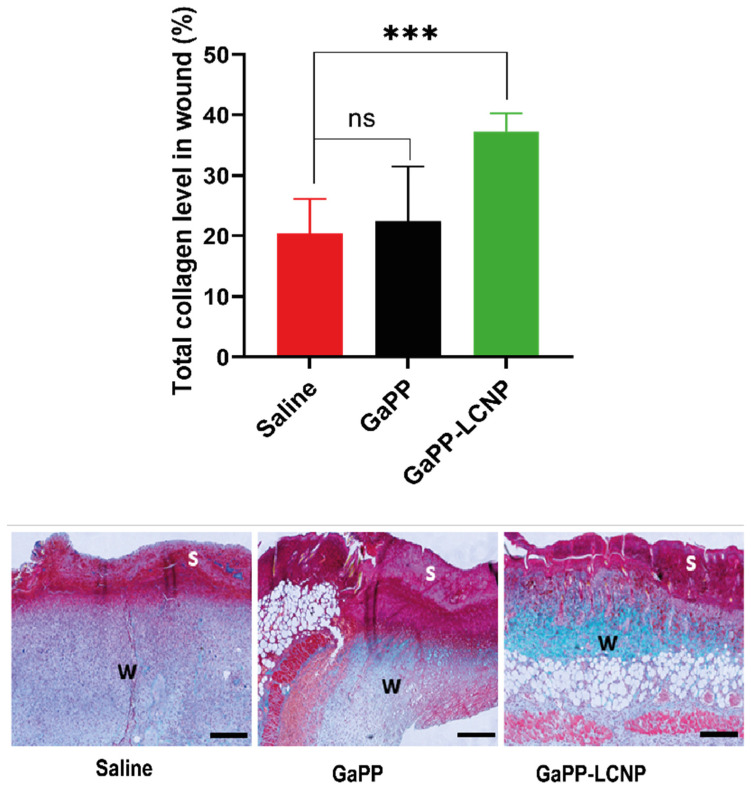
Graphical analysis and representative images of early total collagen deposition in wounds at day six of the in vivo study. The images demonstrate total collagen deposition (green color) in wound bed (w) below the wound scab (s). Data presented as mean ± SD compared to control infected group treated with saline; ns: non-significant, *** *p* = 0.0007 (one-way ANOVA test followed by multiple comparison Dunnett’s test).

In addition to reducing bacterial burden, controlled amounts of ROS have been reported to maintain wound haemostasis and signal platelet aggregation, which stimulates the expression of adhesion molecules [49]. In addition, ROS signals the initiation of the proliferation phase following injury, promoting the expression of basic fibroblast growth factor fibronectin and collagen synthesis [50]. Previously, we have shown that ROS produced by both unformulated GaPP and GaPP-LCNP promoted the proliferation of skin fibroblasts in vitro [17]. Herein, we demonstrate increased early collagen deposition in wounds following the photoactivation process. The GaPP-LCNP group showed significantly higher early collagen content in the wound 37 ± 2.9% compared to 22 ± 9% for unformulated GaPP and 20 ± 5.7% for saline groups, respectively, see Figure 12. This is in agreement with previous studies showing the beneficial effects of aPDT on collagen deposition [51]; however, further studies are needed to elucidate the long term effect at later timepoints of tissue regeneration and scarring. 

The lower performance of unformulated GaPP in vivo is believed to be ascribed to the lower photodynamic activity, wherein the amount of generated ROS is not sufficient to reduce bacterial burden or promote healing, as previously reported in vitro [17]. On the other hand, GaPP-loaded LCNP produced enough ROS to reduce bacterial activity and promote healing of infected wounds. Further research is needed to elucidate the exact mechanism and determine if observed effects on tissue healing and regeneration are secondary to GaPP-LCNP’s effects on bacterial clearance. Interestingly, GMO-based LCNP has recently been shown to promote the healing of non-infected wounds caused by laser burns through helping maintain wound homeostasis [52]. Taken together, the high antibacterial activity of GaPP-LCNP demonstrated in this study, combined with the high safety profile and positive impact on wound healing, justifies further pre-clinical studies using higher light doses to disclose the full potential of GaPP-LCNP as a promising treatment for infected chronic wounds. 

## 4. Conclusions

The potential of GaPP-LCNP as an adjunct treatment for infected chronic wounds was demonstrated in pre-clinical models. The significant in vitro antibiofilm activity (7 log_10_ reduction in bacterial cell viability) of GaPP-LCNP was established against bioluminescent *S. aureus* Xen29 and compared to the previously tested *S. aureus* strains to elucidate the difference in bacterial response to GaPP-LCNP. In addition, the study elaborated the importance of thorough optimization of treatment parameters in ex vivo models before testing in vivo, as the effective light dose and GaPP concentration in vitro had to be significantly increased to reduce the biofilm’s viability by 5.5 log_10_ ex vivo. Most importantly, GaPP-LCNP significantly reduced the viability of biofilms in a cutaneous *S. aureus*-infected wound model by 1 log_10_, while unformulated GaPP could not reduce bacterial wound burden. Moreover, GaPP-LCNP significantly reduced wound area by 90%, and promoted early collagen deposition by ~40%. Future studies on animal models, using a higher number of mice, different light doses and combined therapy with antibiotics are justified to explore the full potential of GaPP-LCNP as a promising treatment for the management of infected chronic wounds. 

## Figures and Tables

**Table 1:** Physicochemical characterization of nanoformulation.

Sample	z-Average Diameter (nm)	Polydispersity Index (PDI)	Zeta Potential (mV)	EE%	DL W/W%
Blank LCNP	182 ± 2.3	0.16 ± 0.03	−23.6 ± 1.2		
GaPP-LCNP	178 ± 3.1	0.19 ± 0.04	−30.5 ± 2.2	98.3 % ± 4.3	3.3 ± 0.3

## Data Availability

Not applicable.

## Supplementary Materials

The following supporting information can be downloaded at: https://www.mdpi.com/article/10.3390/pharmaceutics15020305/s1, Figure S1. Calculated mean fluorescence intensity of GaPP at 585 nm in confocal images of S. aureus and P. aeruginosa biofilms. Fluorescence intensity was calculated using Image J software, *n* = 3; Figure S2. The viability of Xen 29 biofilms collected from infected mouse skin wound after treatment with GaPP in LCNP 15 µM and 50 µM activated with blue light for 2 min, total energy fluence of 16 J/cm^2^ compared to untreated control group. Data presented as mean ± SD, *n* = 3.
