# Supplementary material for: Lipid Liquid Crystal Nanoparticles: Promising Photosensitizer Carriers for the Treatment of Infected Cutaneous Wounds
Source: Pharmaceutics. 2023 Jan 17;15(2):305. doi: 10.3390/pharmaceutics15020305 (PMC9964009; doi:10.3390/pharmaceutics15020305)
Supplement: Supplementary file 1 [file pharmaceutics-15-00305-s001.zip › pharmaceutics-2114974-supplementary.pdf]

Supplementary materials

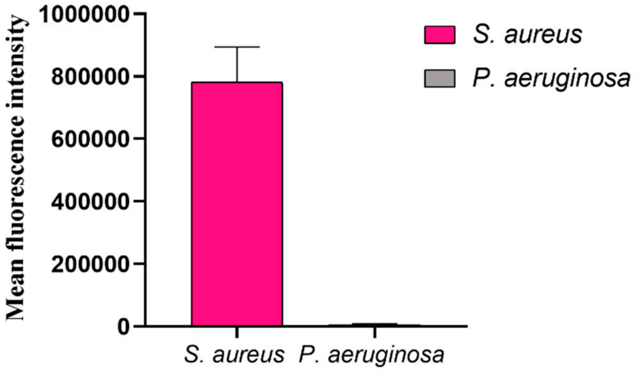

Figure S1. Calculated mean fluorescence intensity of GaPP at 585 nm in confocal images of S. aureus and P. aeruginosa biofilms. Fluorescence intensity was calculated using Image J software, n= 3.

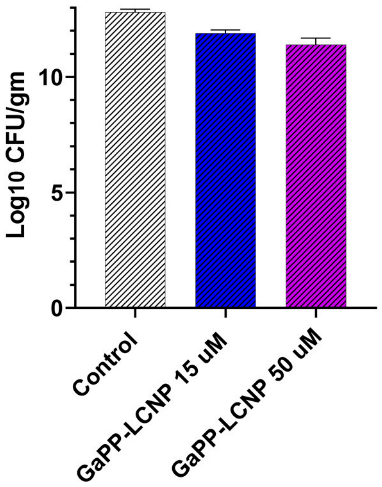

Figure S2. The viability of Xen 29 biofilms collected from infected mouse skin wound after treatment with GaPP in LCNP 15 µM and 50 µM activated with blue light for 2 min, total energy fluence of 16 J/cm^2 compared to untreated control group. Data presented as mean ± SD, n =3.
